# Risk Stratification in People with Diabetes for Fasting During Ramadan: Consensus from Arabic Association for the Study of Diabetes and Metabolism

**DOI:** 10.2174/0115733998249793231005105724

**Published:** 2024-04-05

**Authors:** Inass Shaltout, Amr Mahmoud Abdelwahab, Amr El Meligi, Hany Hammad, Shereen Abdelghaffar, Atef Elbahry, Nasser Taha, Nehal Hamdy Elsaid, Amr Gad, Laila Hammouda, Shaymaa Abdelmaboud, Amin Roshdy Soliman

**Affiliations:** 1 Internal Medicine and Diabetes Department, Cairo University, Cairo, Egypt;; 2 Internal Medicine and Diabetes Department, Minia University, Minia, Egypt;; 3 Internal Medicine and Nephrology Department, Cairo University, Cairo, Egypt;; 4 Department of Pediatrics, Pediatric Diabetes and Endocrinology Unit, Cairo University, Cairo, Egypt;; 5 Cardiology Unit, Port Fouad Centre, Port Fouad, Egypt;; 6 Cardiology Department, Minia University, Minia, Egypt;; 7 Vascular Surgery Department, Cairo University, Cairo, Egypt;; 8 Ophthalmology Department, Minia University, Minia, Egypt;; 9 Cardiology Department, National Heart Institute, Giza, Egypt

**Keywords:** Diabetes, type 2 diabetes, type 1 diabetes, fasting, ramadan, risk stratification

## Abstract

**Background:**

Current international guidelines recommend a pre-Ramadan risk assessment for people with diabetes (PwDM) who plan on fasting during the Holy month. However, a comprehensive risk assessment-based recommendation for the management of PwDM intending to fast is still controversial. Therefore, the Arabic Association for the Study of Diabetes and Metabolism (AASD) developed this consensus to provide further insights into risk stratification in PwDM intending to fast during Ramadan.

**Methods:**

The present consensus was based on the three-step modified Delphi method. The modified Delphi method is based on a series of voting rounds and in-between meetings of the expert panel to reach agreements on the statements that did not reach the consensus level during voting. The panel group comprised professors and consultants in endocrinology (both adult and pediatric). Other members included experts in the fields of cardiovascular medicine, nephrology, ophthalmology, and vascular surgery, affiliated with academic institutions in Egypt.

**Result:**

In PwDM who intend to fast during Ramadan, risk stratification is crucial to optimize patient outcomes and prevent serious complications. The present consensus provides risk assessment of those living with diabetes according to several factors, including the type of diabetes, presence, and severity of complications, number of fasting hours, and other socioeconomic factors. According to their risk factors, patients were classified into four categories (very high, high, moderate, and low risk).

**Conclusion:**

Future research is warranted due to the controversial literature regarding the impact of fasting on certain comorbidities.

## 1 INTRODUCTION

Fasting in Ramadan represents an essential practice in Islam, which is strictly followed by the vast majority of the Muslim population. Due to the increased risk of acute complications, fasting during Ramadan may be challenging for people with diabetes mellitus (PwDM) and their health care providers (HCPs) [1]. Several guidelines have been published for diabetes management during Ramadan. However, most available recommendations/guidelines represent expert opinions rather than evidence-based statements [1-3].

Risk stratification is the cornerstone for guiding PwDM on whether to fast or not. Despite the plenty of publications related to Ramadan fasting in PwDM, the risk stratification for fasting remains controversial. For example, while the presence of significant microvascular diabetic complications was considered among the high-risk category in ADA/EASD guidelines [1], it was not included in the risk scoring system of the IDF-DAR guidelines except for advanced nephropathy [3]. Moreover, while the presence of hard manual work was considered a very high risk for fasting during Ramadan in ADA/EASD guidelines, it was less estimated in IDF-DAR guidelines [3]. Also, the ADA/EASD guidelines did not consider clustering multiple risks in one person. Meanwhile, the IDF-DAR guidelines allowed individualization according to the presence of various risk factors [3].

Regional considerations, although very important, have not been included in the previous guidelines, such as the effect of climate and socioeconomic factors on fasting ability. Climatic conditions can have a substantial influence on the outcomes of PwDM. In the case of extremely hot weather and high humidity, PwDM are at increased risk of morbidity and mortality, particularly among patients with cardiovascular complications [4]. Many countries have high temperatures during the summer period [5]. Some countries, for example, Egypt, have been characterized in recent years by high temperatures in summers, approaching 36-37°C in the center-north and 41-42°C in the south. A temperature as high as 50°C has been reported on some days in the southern part of Egypt [6]. Additionally, the higher annual risk of hypoglycemia in insulin-treated Egyptians with diabetes mellitus (DM) in comparison to other countries, both in type 1 (T1D) and type 2 diabetes (T2D), is another risk factor that needs to be taken into account [7]. Moreover, because of financial obstacles, among other factors, many Egyptians with diabetes do not adhere to self-monitoring blood glucose (SMBG) [8]. Lastly, Egypt, among some other Middle East countries, has a cardiovascular disease (CVD) mortality rate of over 350/100,000 patients, and this high-risk chart may underestimate the risk of fasting [9].

The comprehensive risk assessment-based recommendations for PwDM during Ramadan fasting are still controversial. For the above-mentioned reasons, the Arabic Association for the Study of Diabetes and Metabolism (AASD) developed this consensus to provide recommendations for risk stratification in PwDM intending to fast during Ramadan.

## 2 METHODS

### 2.1 Study Design and Panel Recruitment

The present consensus was based on the three-step modified Delphi method [10]; the modified Delphi method is based on a series of voting rounds and in-between meetings of the expert panel to reach agreements on the statements that did not reach the consensus level during voting. The panel consisted of 12 experts, who were consultant endocrinologists, cardiologists, vascular surgeons, nephrologists, and ophthalmologists and affiliated with academic institutions from Egypt. A non-probability convenient selection process was employed to recruit the experts, ensuring a geographical representation of major academic institutions in Egypt. All panel members were required to sign a disclosure statement before consensus development.

### 2.2 Literature Review and Statements Development

The AASD recruited a survey development committee for the expert panel to develop the initial statements for the first round of voting. The pre-voting statements were developed following a bibliographic search on PubMed using the following MeSH terms: (“Diabetes Mellitus”[MeSH Terms] OR “diabetes mellitus, type 1” [MeSH Terms] OR “diabetes mellitus, type 2” [MeSH Terms] OR “Type 1 Diabetes” OR “Type 2 Diabetes” OR “Diabetes Mellitus Type 2” OR “Diabetic Ketoacidosis”) AND (“Ramadan” OR “fasting”). The evidence was retrieved from guidelines, consensus, systematic reviews, and level 1 quality of evidence studies, as classified by Wright *et al*. [11]. Only studies that were published in the English language were retrieved. Based on the literature search findings, a survey framework with the following domains was developed and covered by a set of statements.

− Risk stratification system of PwDM who intend to fast during Ramadan.
− Risk stratification and recommendations for PwDM and CVD, cerebrovascular disease, and peripheral arterial disease (PAD).
− Risk stratification and recommendations for PwDM and other micro and macrovascular complications.
− Risk stratification and recommendations for older PwDM and other patient populations according to glycemic profile and medications.

### 2.3 Delphi Process and Consensus Development

The initial survey consisted of ternary statements (agree/neutral/ disagree), and the experts could abstain from voting on each statement. A consensus was defined as an agreement level ≥ 80% [12]. Following the first round of voting, the expert panel meeting was held to present the voting results and gather experts' insights on statements that did not reach the consensus level. Accordingly, the second round of votes was held, and the revised statements were sent out to the experts. The final consensus statements and manuscript were reviewed and approved by all panel members.

### 2.4 Risk Stratification Classes of PwDM who Intend to Fast during Ramadan

In PwDM having comorbidities, fasting can pose several challenges, as detailed in the below section. However, there is a lack of universal consensus on the appropriate stratification of PwDM having comorbidities to guide the management recommendations during Ramadan. In the updated IDF-DAR [3], PwDM intending to fast during Ramadan were classified according to the three-tier traffic light system. This system classified patients according to several factors, including length of fasting, type and duration of diabetes, medications, history of hypoglycemia, and diabetic complications. Besides, a set of guidance was provided for patients with CVD and chronic kidney disease (CKD). However, the guideline did not account for other comorbidities or provide risk stratification for each CVD/CKD-related condition [3]. It has also been noted that not all PwDM are at risk of hazardous effects from fasting; for example, the DARMENA T1D study showed that nearly half of the patients who fasted during the full month of Ramadan suffered from hypoglycemia. The percentage was even lower in the DARMENA T2D study [13,14].

Therefore, the experts agreed that the risk stratification of PwDM should account for comorbidities and their impact on the risk of hypoglycemia, as comorbidities may exacerbate the risk of hypoglycemia, while impaired glycemic control may fasten the progress of these complications. The panel emphasized the need for a detailed explanation of the possible risks to the patients and recommendations according to their risk group. The experts agreed on four categories for risk stratification of PwDM and comorbidities, according to the nature and number of risk factors. Patients with at least two high-risk factors or one high plus three moderate risk factors were classified as very high-risk patients. In the presence of five moderate risk factors, the patients were considered among the very high-risk group, while in the presence of four moderate risk factors, patients were categorized as high-risk (Table **1**).

### 2.5 Risk Stratification for PwDM According to their Management (in T2D)

#### 2.5.1 Oral Therapy

Since Ramadan fasting has a significant potential for several metabolic changes, the risk for hypoglycemia is increased, especially in PwDM commencing on medications. Some classes of medications are associated with higher hypoglycemia and dehydration risks. In return, adequate risk stratification for T2D individuals who intend to fast Ramadan is crucial, and if needed, medication adjustments are required.

##### 2.5.1.1 Metformin

Metformin, when used alone, rarely causes hypoglycemia. Despite the absence of clinical trials evaluating its safety in PwDM planning to fast during Ramadan, it is regarded as a safe medication [3].

##### 2.5.1.2 Sulphonylureas (SUs)

Since SUs act by stimulating insulin secretion from the pancreas independent of blood glucose level, it is associated with an increased risk of hypoglycemia during fasting. Consequently, a major concern related to using SUs during Ramadan has been issued. Nonetheless, the risk of hypoglycemia varies among this class of medications due to different duration of action and receptor interactions [3].

In a multinational observational study, Aravind *et al.* included 378 individuals with T2D who fasted during Ramadan while being treated with SUs and reported that 20% of the included patients experienced hypoglycemic episodes with the highest rate among patients receiving glibenclamide than glimepiride and gliclazide [15]. Similarly, the VIRTUE study comparing vildagliptin with SU among T2D individuals during Ramadan reported a higher incidence of hypoglycemia (31.8%) among glibenclamide-treated people compared to gliclazide arm (19.2%) or glimepiride arm (17.9%) [16].

More recently preferred SU medications have a more manageable safety profile regarding the rate of hypoglycemia during Ramadan. In the STEADFAST trial, the incidence of hypoglycemia was lower in the gliclazide groups (7.0%) [17]. Moreover, a multinational observational study reported a low incidence of hypoglycemia in people with T2D using glimepiride during Ramadan fasting, and the rate did not differ significantly before and after Ramadan [18]. Recently, DIA-RAMADAN, a large real-world international study, included 1244 adults with T2D treated with gliclazide-modified release (MR). The results showed that the incidence of symptomatic hypoglycemia during Ramadan was low (2.2%) without the report of severe hypoglycemia [19]. In light of the above literature, people with T2D who use gliclazide, glimepiride, and gliclazide MR have a low risk of hypoglycemia and these medications can be used safely during Ramadan.

##### 2.5.1.3 Thiazolidinediones (Pioglitazone)

The hypoglycemic risk is generally low in PwDM receiving pioglitazone as it does not exert its glycemic effect via stimulating insulin secretion [20]. Vasan *et al*. conducted an RCT in India to evaluate the effect of pioglitazone during Ramadan on 86 fasting Muslims. The trial reported pioglitazone to be associated with glycemic parameter improvements at the time of fasting and after it. Moreover, the incidence of hypoglycemia was comparable between pioglitazone and placebo groups [21].

##### 2.5.1.4 Alpha-glucosidase Inhibitors

The risk of hypoglycemia in individuals using these medications is low. In addition, there is no published clinical trial that has assessed the impact of using alpha-glucosidase inhibitors on fasting during Ramadan. Based on that, PwDM receiving alpha-glucosidase inhibitors can fast safely during Ramadan [3].

##### 2.5.1.5 Prandial Glucose Regulators (Glinides)

Many studies have reported a low-to-moderate risk of hypoglycemic episodes in individuals treated with repaglinide during Ramadan [22-24]. Mafauzy reported the incidence of hypoglycemic episodes in an open-label randomized trial comparing hypoglycemic episodes in patients receiving repaglinide *vs*. glibenclamide during Ramadan as 2.8% *vs*. 7.9%, respectively [24]. On the other hand, Anwar *et al.* compared repaglinide vs. glimepiride and found similar symptomatic hypoglycemic events during Ramadan in both the groups [25].

##### 2.5.1.6 Dipeptidyl Peptidase-4 (DPP-4) Inhibitors

DPP-4 inhibitors are well-tolerable oral anti-diabetic drugs (OAD), demonstrating a minimal risk of hypoglycemia in non-fasting patients. A number of trials and observational studies have evaluated the safety of DPP-4 inhibitors in patients who intend to fast during Ramadan. In a large trial of 1,066 individuals with T2D who switched to sitagliptin from SUs and fasted during Ramadan, the risk of hypoglycemia was lower upon switching to sitagliptin than with continuing SUs [26]. Likewise, a trial reported a reduction in hypoglycemia risk with a sitagliptin regimen compared to SUs in patients who observed Ramadan fasting [27]. In the STEADFAST study, which included 557 patients with T2D taking either vildagliptin or gliclazide during Ramadan fasting, the risk of hypoglycemia was lower with vildagliptin than with gliclazide [17]. Likewise, in the observational VECTOR and VIRTUE studies, vildagliptin was associated with a lower frequency of hypoglycemic events than SUs [28, 29].

In a previous meta-analysis, it was demonstrated that PwDM on DPP-4 inhibitors, who fasted during Ramadan, had a lower frequency of hypoglycemia, including severe forms of hypoglycemia, than SUs [30]. A multicenter observational study in Egypt (EMPA-Ramadan) has reported both DPP-4 inhibitors and SGLT-2 inhibitors to lead to a low incidence of hypoglycemia, and that they can be used safely in T2D management during Ramadan [31].

##### 2.5.1.7 Sodium-glucose Co-transporter-2 (SGLT-2) Inhibitors

Despite their well-established efficacy, some safety concerns are raised among SGLT-2 inhibitor users who intend to fast during Ramadan. SGLT-2 inhibitors may increase the risk of dehydration in at-risk groups, such as those on loop diuretics, a concern that may be aggravated particularly during Ramadan [32].

A few studies have evaluated the safety of SGLT-2 inhibitors during Ramadan. Wan Seman *et al.* found a lower risk of hypoglycemia with dapagliflozin than SUs in PwDM fasting in Ramadan, with a non-significant increase in the incidence of postural hypotension and urinary tract infections (UTI) [33]. Likewise, the CRATOS study demonstrated a reduction in the hypoglycemia risk associated with the use of SGLT-2 inhibitors for T2D patients fasting during Ramadan [34]. Interestingly, Abdelgadir *et al.* used flash glucose monitoring to assess the safety of SGLT-2 inhibitors during Ramadan. The results showed a low risk of hypoglycemia during fasting with no reported adverse events [35]. In light of these studies, people with T2D receiving SGLT-2 inhibitors should be on these drugs at least four weeks before Ramadan fasting to avoid possible complications.

#### 2.5.2 Summary of Recommendations

- Patients on treatment with diet and other lifestyle measures are considered a low-risk group and can fast safely during Ramadan.
- PwDM receiving metformin, alpha-glucosidase inhibitors, thiazolidinediones, and DPP-4 inhibitors are categorized as low-risk and can fast safely during Ramadan.
- People with T2D using second-generation SUs and glinides are considered among the moderate-risk group and can fast with medical assistance, except for patients using gliclazide MR are considered among the low-risk group and can fast safely during Ramadan. Glibenclamide should be changed for PwDM who intend to fast during Ramadan.
- Patients on SGLT-2 inhibitors are considered among the low-risk group and can fast safely during Ramadan, provided that they are started at least four weeks before Ramadan.

#### 2.5.3 Injectable Therapy

##### 2.5.3.1 Insulin

The major life-threatening risk caused by the use of insulin is hypoglycemia. Insulin therapy represents a challenge for both physicians and PwDM who intend to fast during Ramadan [2].

- Basal insulin (BI): It has been demonstrated that glargine U100 (Gla-100) and detemir pose a reduced risk of hypoglycemia than NPH. Glargine U300 (Gla-300) and degludec are linked to a decreased incidence of hypoglycemia than glargine Gla-100. In the DEVOTE Trial, insulin degludec was linked with a markedly reduced risk of hypoglycemia compared to Gla-100 [36]. In the ORION study, Gla-U300 therapy was associated with a low incidence of severe hypoglycemia [37].

According to a multinational study, people with T2D treated with insulin glargine and glimepiride experienced a higher rate of mild hypoglycemic episodes during Ramadan compared to pre and post-Ramadan [38].

- Premixed insulin and basal-bolus regimen: Premixed insulin/insulin analogue regimens are simple and attractive for many physicians and patients. Fixed meal timing and activity level are required, and are associated with more weight gain and hypoglycemia than the basal-bolus regimen [39]. Basal bolus regimen (MDI) mimics the physiological pattern of insulin secretion and provides flexibility in food intake.

The safety and efficacy of biphasic insulin analogues versus premixed human insulin 30/70 in people with T2D were evaluated in real-world settings before, during, and after Ramadan fasting. During Ramadan, Matto *et al.* found improved average daily glycemic and BG control with insulin lispro Mix25 before and after the evening meal [40].

Soewondo *et al*. showed that biphasic insulin aspart 30 (BIAsp 30) significantly reduced all glycemic parameters. After three months, BIAsp 30 reduced hypoglycemic episodes more than premixed human insulin [41].

In a multinational, open-label, randomized, controlled trial, insulin degludec/insulin aspart was compared to BIAsp in individuals with T2D prior to, during, and four weeks after Ramadan. Compared to BIAsp 30 BID, IDegAsp BID maintained comparable glycemic control before, during, and after Ramadan, with lower rates of overall and nocturnal symptomatic hypoglycemia. The rate of severe hypoglycemia was only numerically lower in the IDegAsp BID arm compared to the BIAsp 30 BID arm.

The authors noted the following study limitations: first, the study design, which was open-label, may result in treatment bias. Second, the fasting hours in this study ranged from 12 to 16 hours and did not include populations from countries with longer fasting hours [42]. Moreover, hypoglycemic episodes with plasma glucose (PG) levels less than 3.1 mmol/L (56 mg/dL) were documented. The authors of this consensus still consider both types of insulin (premixed human and premixed analogues); although safer with the second one, a high risk for fasting in Ramadan exists if taken in more than one injection daily because of the residual risk of severe hypoglycemia, especially in people with T2D who usually have advanced macro and microvascular complications at the time of insulin initiation.

Regarding pre-meal insulins, rapid-acting insulin analogues (RAIA) have immediate onset of action, earlier peak, and shorter duration of action compared to rapid-acting human insulin (RAHI), so they provide better control of postprandial blood glucose with a lower risk of postprandial hypoglycemia [43]. The RAIA, insulin lispro versus RAHI, taken before Iftar in T2D fasted participants in Ramadan, was associated with significantly lower postprandial blood glucose and a lower rate of hypoglycemic episodes [44].

The studies addressing insulin/insulin analogues during Ramadan fasting have many limitations, as follows: many studies are not randomized controlled studies, including a small number of participants, some are open-labelled, and fasting hours are different. These limitations make us unable to correctly estimate the seriousness of fasting in insulin-treated people with T2D diabetes.

##### 2.5.3.2 Summary of Recommendations

- Fasting represents a very high risk in patients treated with multiple daily insulin injections.
- Fasting represents a high risk in patients taking two doses of premixed insulin.
- Fasting represents a moderate risk in patients on stable doses of BI.
- PwDM treated with a single daily dose of premixed insulin before Iftar (both human and analogue) are considered among the moderate risk group and can fast with medical assistance.

#### 2.5.4 Glucagon-like Peptide-1 Receptor Agonists (GLP1-RAs)

GLP1-RAs stimulate insulin secretion by the beta cells in a glucose-dependent manner [45, 46]. Thus, as a monotherapy, the risk of severe hypoglycemia is low, while the risk may be elevated if commenced with SUs or insulin [47]. Nausea and vomiting are the most annoying side effects, especially in the early period after the initiation of GLP1-RAs. Once weekly GLP1-RAs, such as dulaglutide and semaglutide, have not been studied during Ramadanl however, they may provide an attractive option for fasting patients who prefer a simplified regimen [48].

Regarding the safety of GLP1-RAs use, the TREAT 4 Ramadan trial conducted by Brady *et al*. evaluated the safety and efficacy of liraglutide as a part of a diabetes management regimen compared to SU with metformin during Ramadan in people with T2D. The results showed a lower incidence rate of hypoglycemia in patients who received liraglutide than SUs. Furthermore, the liraglutide group showed better glycemic parameters post-Ramadan [49].

Likewise, Khalifa *et al*., who assessed the effectiveness and tolerability of liraglutide as an add-on therapy to existing anti-diabetic medications in Ramadan in 111 individuals, reported no increased risk of hypoglycemia in patients who received liraglutide during Ramadan. The risk of hypoglycemic events was primarily related to the duration of diabetes [50]. Besides, the LIRA-Ramadan trial in Africa and Asia reported better glycemic control and no weight gain with a lower incidence rate of hypoglycemia with liraglutide than SU [51].

Concerning exenatide, an observational study conducted in the UK by Bravis *et al*. among people with T2D who were commencing on exenatide as an add-on therapy to metformin during Ramadan showed no significant changes in the rate of hypoglycemia and body weight [52].

Regarding lixisenatide, Hassanein *et al*. found no risk of hypoglycemic events in the lixisenatide + BI group compared to the SU+BI group during fasting hours [53].

The fixed-ratio combination (FRC) of the GLP1-RAs lixisenatide and Gla-100 (iGlarLixi FRC) was evaluated in the SoliRam trial involving 155 Muslims who intended to fast Ramadan before, during, and after Ramadan, and it has shown significant improvements in glycemic control and body weight between the pre- and post-Ramadan periods. This was associated with a low incidence of hypoglycemia [54].

#### 2.5.5 Summary of Recommendations

- Fasting represents a low risk in patients on stable doses of GLP1-RAs at least 4-6 weeks before Ramadan.
- GLP1-RAs are considered a good choice during Ramadan fasting in obese patients, those with established atherosclerotic cardiovascular disease (ASCVD), or those with high-risk factors for ASCVD who intend to fast during Ramadan.
- FRC (BI+GLP1-RA) with proven safety during Ramadan is considered a moderate-risk group for fasting during Ramadan.

### 2.6 Risk Stratification for People with Special Conditions

#### 2.6.1 Risk Stratification for People with Type 1 Diabetes (T1D)

Most international societies and associations consider T1D a high or very high-risk condition for fasting due to the increased risk of dehydration, hypoglycemia, and hyperglycemia [3, 55, 56]. Moreover, fasting accelerates lipolysis, ketosis, and glucagon levels, which accounts for the increased risk of developing diabetic ketoacidosis (DKA) [57, 58]. Risk stratification for people with T1D who insist on fasting depends on metabolic control before Ramadan, the presence of acute or chronic complications of diabetes, and other comorbidities that increase the risk of fasting [56].

The EPIDIAR observational cohort, which was conducted on 1070 adults with T1D, demonstrated a significant increase in severe hypoglycemia by 4.7-fold during Ramadan [59]. In addition, continuous glucose monitoring (CGM) data demonstrated notable variability in glucose levels, with fluctuations between fasting and non-fasting hours, as concluded by Kaplan and Afandi [60]. Moreover, Afandi *et al.* concluded that CGM data revealed substantial blood glucose level fluctuations during fasting and non-fasting hours [61]. Thereafter, Afandi *et al*. recruited 50 children and adolescents with T1D and found that those with uncontrolled glycemic profiles had a significantly higher frequency of hypoglycemia, particularly before Iftar time, than those with lower HBA1c [62]. Moreover, Alfadhli observed that those with poorly controlled T1D had substantial blood glucose level fluctuations during fasting and non-fasting hours, increasing the risk of hyperglycemia [63].

Hence, poorly controlled people with T1D, with or without the presence of comorbidities or complications, are widely recognized as a very high-risk group who must abstain from fasting during Ramadan, while those well-controlled are recognized as a high-risk group, who should be advised not to fast during Ramadan [13]. For individuals who insist on fasting, frequent SMBG or CGM is advised during fasting to reduce hypo/hyperglycemic episodes and warn the patient to break fasting [55]. In addition, real-time monitoring of blood glucose fluctuations through CGM can help in informing dose adjustment strategies during Ramadan [3]. Besides, as per Benbarka *et al*.’s findings, insulin pumps can provide further support in the dosing adjustment strategy and help in reducing the hypo/hyperglycemia risk [64]. Sensor-augmented pumps (SAP) and appropriate counselling can reduce the risk of complications from fasting [65, 66].

Therefore, patients who insist on fasting should be warned and subjected to pre-Ramadan assessment and risk stratification. They must receive appropriate comprehensive education at least one month before Ramadan about probable complications associated with fasting and appropriate means to deal with them [56]. Individualized Ramadan-specific treatment regimens should be provided for each patient respecting regional and cultural factors [58]. Insulin type, dosage, and timing may need to be modified to minimize possible risks during Ramadan fasting. Patients who insist on fasting should be offered an individualized meal plan to ensure an adequate balanced diet during Ramadan and educated to use proper carbohydrate counting for meals to match the insulin dose [55]. It has to be stressed that people with T1D with good metabolic control using an insulin pump and/or CGM with compliance, strict follow-up, and time-in-range of 70% or more can be classified as moderate-risk and can fast with caution under medical supervision, as previously recommended [55].

##### 2.6.1.1 Summary of Recommendations

- Poorly controlled T1D individuals or those with comorbidities and/or chronic complications are considered a very high-risk group and must not fast during Ramadan.
- Well-controlled T1D individuals are considered among the high-risk group and should not fast during Ramadan.
- Well-controlled T1D individuals using insulin pump and/or CGM are considered a moderate-risk group and can fast with caution under medical supervision.

#### 2.6.2 Ramadan Fasting in Pregnant and Breastfeeding Women with Diabetes

Diabetes during pregnancy represents a significant challenge of tight glycemic control and maintaining the euglycemic state with insulin therapy. Consequently, much effort is necessary to prevent severe hypoglycemic episodes. Hypoglycemia is unavoidable while striving to achieve HbA1c 6%, normal fasting blood glucose (FBG), one-hour post-prandial blood glucose (PPBG), and two-hour PPBG levels, as required in most international guidelines [67].

Pregnancy itself may be associated with a dysfunctional counter-regulation mechanism and a state of hypoglycemic unawareness [68]. The risk of hypoglycemia in pregnant women with DM is mostly attributable to the medication pharmacokinetics (*i.e.,* producing excessively high insulin concentrations) and failure in the physiological protective system that corrects falls in serum glucose concentrations [68, 69]. Approximately 45-71% of women with T1D had severe hypoglycemia during pregnancy [70-72]. Severe hypoglycemia during pregnancy is more common in the first trimester and occurs less frequently in the third trimester [70]. Whether hypoglycemia harms a developing baby is a matter of debate. Its potential effect on teratogenicity, in particular, is still debatable. Also, placental insufficiency presents in the third trimester with unwarranted hypoglycemia, especially if associated with falling insulin requirements [73].

The decreased blood glucose levels in pregnant women may affect fetal outcomes. For example, they may cause fetal growth retardation [74], small for gestational-age infants [75], and impaired fetal β-cell function [76]. The hazardous effect of recurrent hypoglycemia on the adult brain is well-known [77-79]. However, there is no available data on the long-term effects of recurrent hypoglycemia on fetal brain health, whether during intrauterine life or later on. Therefore, we cannot guarantee the safety of maternal fasting during Ramadan for long-term fetal brain health.

A “perfect storm” of metabolic abnormalities, such as hyperglycemia, hypoglycemia, and ketosis, may occur in diabetic pregnant women who fast during Ramadan [80]. There is an underlying pathophysiology of insulin shortage and/or resistance, which may result in increased gluconeogenesis, glycogenolysis, and ketogenesis when PwDM fast because reduced glucose intake suppresses insulin production [81]. This might be worsened during the second and third trimesters of pregnancy, referred to as a time of “accelerated starvation.” In addition, human placental lactogen induces insulin resistance and poor glucose utilization in the mother in order to shift the glucose supply to the fetus. This can result in an abnormally high maternal ketogenesis response to fasting [82, 83]. Therefore, Ramadan fasting in pregnant women with diabetes may represent a “perfect storm” of circumstances that leads to potentially serious metabolic changes, like ketogenesis [80].

Mirghani and Hamud found a higher rate of induction of labour, cesarean section, and admissions to the special care unit of the newborn in the fasted group compared to the control group [84]. Diabetes or gestational DM (GDM) during pregnancy increases the risk of unfavourable maternal and fetal outcomes; therefore, women with diabetes who become pregnant are instructed to avoid Ramadan fasting [1].

Regarding breastfeeding, data suggest that women with diabetes may experience hypoglycemic events during lactation; therefore, they are advised to eat before or during breastfeeding to avoid hypoglycemia [85]. Achieving desired metabolic control in fasting lactating women during Ramadan is challenging.

To the best of our knowledge, no previous studies have investigated the impact of Ramadan fasting on diabetic breast-feeding women. Therefore, we consider this point a knowledge gap, which we recommend being studied in future research.

##### 2.6.2.1 Summary of Recommendations

- Pregnant ladies with diabetes, whether T1D, T2D, or gestational diabetes, are considered a very high-risk group and must not fast during Ramadan.
- Lactating women with DM who are not taking insulin are considered a moderate-risk group and may fast during Ramadan with medical supervision.
- Lactating women with DM on insulin therapy are considered a very high-risk group and must not fast during Ramadan.

#### 2.6.3 Risk Stratification of Ramadan Fasting in Elderly PwDM

Advances in medical care and improved social conditions have increased life expectancy, and the epidemic of diabetes may involve older individuals. According to IDF 9^th^ Atlas, 1 in 5 PwDM is above 65 years old, with a total of 163 million people [86].

Diabetes in old age is a risk factor for dementia, falls, hip fractures, CVD, amputation, and visual impairment [87]. Elderly PwDM have an increased risk of volume depletion [88]. Thus, fasting during Ramadan in this group may affect their postural balance and attention, which may increase the risk of falls or fall-related injuries [89, 90].

The risk of hypoglycemia is increased in the elderly and may manifest with neuroglycopenic features, such as dizziness, delirium, and confusion. Severe hypoglycemia accounts for 17% of all hospitalizations in the elderly with T2D. Drugs increasing the risk of hypoglycemia, including beta blockers, salicylates, warfarin, and tricyclic antidepressants, are better avoided in the elderly with diabetes [91].

Age per se should not be a barrier to safe fasting during Ramadan. Comorbidities, complications, type of antidiabetic medication, and the presence of hypoglycemia or hypoglycemia unawareness will remain the main concern. The presence of CVD and impaired renal function could compromise the safety of fasting in the elderly with longstanding diabetes, making their glycemic control challenging [92].

In the recently published DAR Global Survey on individuals with T2D during Ramadan 2020, 28.5% of participants above the age of 65 fasted despite having HbA1c above 9%. Around 20% of participants above 65 years had coronary artery disease (CAD) and stroke, while 66% were hypertensive. A greater proportion of elderly participants, *i.e*., 69%, completed 30 days of Ramadan fasting compared to 60% of those below 65 years of age. The number of fasting days was similar in both age groups. 17% of participants above 65 years old experienced more hypoglycemic attacks than younger participants. One in every five insulin users above 65 years showed more prevalence of hypoglycemia and hyperglycemia [93].

Pooled safety and efficacy analysis of 10 randomized, double-blind studies on 12,326 candidates who used vildagliptin showed no hypoglycemia, and HbA1c reduction was 1.1% in elderly above 75 years, so the use of DPP-4 inhibitors seems to be attractive in the elderly because there is no risk of hypoglycemia [94].

Dapagliflozin was studied in age groups <65, 65-75, and >75 years; the frequency of overall adverse events (AEs) was slightly higher *versus* placebo. UTI, genital infections, and a few instances of volume depletion were higher in all age groups [95].

##### 2.6.3.1 Summary of Recommendations

- Frail older people are categorized as very high risk and must not fast during Ramadan.
- People with dementia and cognitive disorders are among the very high-risk groups and must not fast during Ramadan.
- Older people with poor health or associated comorbid conditions are categorized as high-risk groups and should not fast during Ramadan.
- Older people living alone on insulin or old-generation SUs are categorized as high-risk groups and should not fast during Ramadan.
- Older people above 75 years and not having any other comorbidities are categorized as a moderate-risk group and may fast in Ramadan with caution.
- Recommendations for safe fasting in the elderly:
  - Pre-Ramadan-focused diabetes education on safe fasting and when to break the fast in conditions of hypoglycemia, hyperglycemia, or dehydration, and to improve hypoglycemia awareness.
  - Stress on home blood glucose monitoring.
  - Good hydration, especially in summer with longer fasting duration.
  - DPP-4 inhibitors should be used over SUs as second-line therapy after metformin.
  - When using SUs, gliclazide MR and glimepiride should be used instead of glibenclamide.
  - Meglitinides may be used instead of glyburide.
  - Basal insulin analogues may be used instead of NPH.

### 2.7 Risk Stratification in PwDM and Acute Diabetic Complications

Serious complications may occur during fasting in Ramadan in PwDM, mostly hypoglycemic coma, hyperglycemia, DKA, and hyperosmolar hyperglycemic state (HHS). Improper dose adjustment of treatment, lifestyle changes during Ramadan, and physical activities are the contributors to these complications. Hyperglycemia may result from reducing the dosage of antidiabetic drugs and increased food or sugar intake. DKA risk increases due to inappropriate insulin dosage reduction, particularly in those with T1D who fast. Uncontrolled PwDM before Ramadan fasting, those with renal insufficiency, advanced micro and macrovascular complications, or other comorbidities are at increased risk of developing DKA or HHS [96].

For the above-mentioned causes, severe hypoglycemia three months before Ramadan, recurrent hypoglycemia, or hypoglycemia unawareness are considered very high risk. Furthermore, those having DKA or HHS 3 months before Ramadan are also considered very high risk [1].

#### 2.7.1 Summary of Recommendations

- PwDM and hypoglycemia unawareness and recurrent or unexplained hypoglycemia in the last three months before Ramadan are considered a very high-risk group and must not fast.
- PwDM and DKA in the last three months before Ramadan are considered a very high-risk group and must not fast.
- PwDM and hyperosmolar coma in the last three months before Ramadan are considered a very high-risk group and must not fast.

### 2.8 Risk Stratification in Patients with Macrovascular Complications

#### 2.8.1 Risk Stratification for PwDM and CVD who Intend to Fast during Ramadan.

The association between ASCVD and poor glycemic indices is well established. PwDM have an elevated risk for ASCVD and are associated with a worse prognosis compared to the normal population [97-99]. Furthermore, suboptimal glycemic control contributes significantly to elevated CVD risk among the diabetic population. People with T2D without a history of myocardial infarction (MI) had a similar risk of cardiovascular death as those with a history of MI but no diabetes. Also, patients with both T2D and a history of MI had a risk of cardiovascular death that was nearly three times that of individuals with a history of MI but no diabetes [100]. Moreover, in the recently published European Study of Cardiology Guidelines, Egypt, among other countries, is categorized as a very high-risk country that has a cardiovascular mortality rate of more than 350/100,000 [101].

Although there has been a lot of published data on the benefits of fasting for healthy adults, there is a dearth of clinical data on how Ramadan fasting affects those who are unhealthy. Although there are guidelines for safe fasting for people with certain chronic illnesses, such as DM, clinical practice recommendations for DM patients with CVD are still unclear. Due to the scarcity of clinical trials, clinical practice guidelines, or expert consensus, HCPs are unable to advise their CVD patients on whether or not to fast during Ramadan. All PwDM who are willing to fast during Ramadan should be educated about the risks of fasting (such as dehydration and fluid overload) and the management of possible complications (including fasting termination if they become unwell). Also, they should undergo a clinical evaluation following Ramadan to reevaluate their risk status and overall health condition [102].

During Ramadan, the long duration of fasting results in an incomplete adherence to the medications and missing several therapeutic doses [103-105]. Also, medication non-compliance is an important issue for PwDM and CVD, especially during Ramadan fasting, which can be life-threatening [106]. In addition, some of these medications are taken twice daily, which is difficult to achieve during Ramadan. Besides, inappropriate nutritional habits, an increased carbohydrate diet, salt intake, less hydration, sleep disturbance, and lack of activities might negatively affect the risk of CVD in PwDM [107, 108]. Unfortunately, research data on the effects of fasting during Ramadan for people with cardiomyopathies, arrhythmias, or valvular heart disease, are limited.

DM can increase mortality and complications in patients who have recently experienced acute coronary syndrome (ACS) [109]. A recent study recommended that patients within six weeks of ACS are advised not to fast [102]. Meanwhile, other authors recommend against fasting in patients with recent percutaneous cardiac intervention (PCI) (within three months) due to the risk of stent thrombosis from dehydration [110]. However, the authors of this article consider this as an underestimation of the situation, and those patients should wait for six months before the decision to fast is taken.

Thrombogenicity, aggravated by dehydration, is known to promote late stent thrombosis. Due to the potential dehydration risk, Ramadan fasting after a recent PCI may increase the risk of stent thrombosis; hence, many cardiologists advise against it [110]. Although there are no consensus guidelines, it looks reasonable to advise patients with CVD (such as MI, unstable angina, or recent revascularization) to avoid Ramadan fasting [111].

As regards chronic coronary syndrome (CCS), a few studies have found no increase in morbidity or mortality in these patients during Ramadan fasting [112, 113]. However, these studies were small in sample size, observational, and included patients mostly without diabetes. In addition, other factors, such as increased thrombogenicity [110], drug resistance [114], dosing change [115], and medication adherence [103, 104] can increase CV events during fasting. Some experts suggest that CCS and heart failure with reduced ejection fraction (HFrEF) patients could safely fast throughout Ramadan with close monitoring [113,116]. Ramadan fasting evidence is scarce for some CVDs, such as cardiomyopathies, arrhythmias, and valvular heart disease. Observational study design, small sample size, and lack of post-Ramadan follow-up limit Ramadan fasting recommendations. There is a lack of a clear definition of “close observation” or specific information on patients' diet outside of fasting.

As regards hypertension (HTN), in an observational study on 15 hypertensive patients, investigators found a statistically significant reduction in systolic and diastolic blood pressure (BP) after Ramadan fasting. Notably, all included subjects had mild HTN and continued their antihypertensive therapy during Ramadan [117]. Another large meta-analysis that investigated the effect of Ramadan fasting on BP found Ramadan fasting as associated with lower BP in patients with HTN as well as subjects with DM [118]. Habbal and his colleagues also supported Ramadan fasting for patients with non-complicated essential HTN [119]. To our knowledge, no previous studies have investigated the impact of Ramadan fasting on hypertensive PwDM. To avoid the risk of worsening kidney function, HCPs should reevaluate BP, kidney function, and hypertensive medications with a diuretic component, such as aldosterone antagonists or thiazide-like diuretics, during a regular visit before Ramadan fasting. Also, home BP monitoring will help to track the patient’s BP response to antihypertensive therapy during Ramadan fasting [120].

Ramadan fasting in PwDM taking antithrombotic therapy is an important challenge for both physicians and patients. Fasting dramatically lowered platelet sensitivity to clopidogrel in PwDM. This impact may be a result of fasting-induced increases in glucose and serum lipids [121]. Additionally, aspirin resistance was also reported to be increased in PwDM fasting during Ramadan, an effect that persisted for one month after Ramadan [114]. Moreover, non-compliance with antiplatelet therapy in patients who have undergone coronary stent implantation can be fatal [122].

In a cohort of 809 patients on oral anticoagulants and fasting during Ramadan, the investigators found that 50% of the patients modified their anticoagulation regimen without consulting their treating physician (due to causes including changing the dosing schedule, skipping doses, or drugs being taken at the same time two times per day) [115]. Also, patients on twice-daily direct oral anticoagulants were observed to be more likely to change their anticoagulation drugs and be hospitalized as a result of these modifications due to causes, such as bleeding or stroke [115]. Published evidence on the impact of Ramadan fasting on patients with atrial fibrillation (AF), inherited arrhythmic syndromes, valvular heart disease, prosthetic valves, pulmonary HTN, heart transplantation, or adult congenital heart disease, is limited.

The impact of fasting on cardiac arrhythmia is rarely examined. A retrospective study found no correlation between fasting time and cardiac arrhythmia hospitalization in ASCVD patients; the same was true in the subgroup of patients with ASCVD [123]. Another report by Al Suwaidi *et al*. showed similar findings [108]. Furthermore, Hyltén-Cavallius *et al.* reported that in patients with long-QT syndrome type 2, there was an increase in glucagon-like peptide1, gastric inhibitory polypeptide, and insulin secretion with a defect in glucagon secretion, resulting in a decrease in plasma glucose and an increased risk of hypoglycemia [124]. Also, hypoglycemia could prolong the QT interval and increase the risk of cardiac arrhythmia [125, 126]. Therefore, PwDM and long-QT syndrome must avoid Ramadan fasting.

Concerning heart failure (HF), an observational study that utilized data from Gulf CARE (Gulf acute heart failure registry) found no significant difference in hospitalization rate due to HF in PwDM during Ramadan [127]. Moreover, Abazid *et al.* studied the Ramadan fasting effect on 249 patients with chronic HF. The study showed that patients who were non-compliant with the medical treatments and dietary recommendations had hemodynamic instability with progressive symptoms of HF [116].

#### 2.8.1.1 Summary of Recommendations

- PwDM and recent ACS (six months before fasting) are considered a very high-risk group and must not fast during Ramadan.
- PwDM and recent revascularization (PCI or CABG) within the last six months before Ramadan are at a very high risk and must not fast during Ramadan.
- PwDM and CCS are considered a high-risk group and should not fast during Ramadan.
- PwDM and stable ASCVD are considered a high-risk group and should not fast during Ramadan.
- PwDM and severe valvular heart disease are considered a very high-risk group and must not fast during Ramadan.
- PwDM and uncontrolled HTN or mild-to-moderate valvular heart disease are considered a moderate-risk group and may fast under medical supervision.
- Patients with hypertensive urgency or emergency are considered a very high-risk group and must not fast during Ramadan.
- PwDM and cardiac arrhythmia are considered a very high-risk group if they have poorly controlled arrhythmia or are treated by anticoagulants for stroke prevention in AF.
- PwDM and the long-QT syndrome are considered a very high-risk group and must not fast during Ramadan.
- PwDM and severe congestive HF, acute HF, or ejection fraction <35% are considered a very high-risk group and must not fast during Ramadan.
- PwDM and prosthetic heart valves are considered a very high-risk group and must not fast during Ramadan.
- For safe fasting, medications taken twice daily should be shifted to other alternative medications (with the same mechanism of action) that are taken once daily.

#### 2.8.2 Risk Stratification in PwDM and Cerebrovascular Disease

DM is a known risk factor for ischemic cerebrovascular events. The impact of Ramadan fasting on the risk of cerebrovascular complications in PwDM is debatable. Comouglu *et al.* reported an increase in stroke incidence among PwDM fasting during Ramadan but a decrease in the risk of hemorrhagic stroke among HTN patients; they attributed this to an increase in hyperglycemia [128]. In another study involving 22 PwDM among 60 patients, researchers noted Ramadan fasting as an independent risk factor for ischemic stroke, which they attributed to the hot weather and long fasting hours [129]. In contrast, there was no statistically significant increase in the number of ischemic strokes among T2D individuals who fasted during Ramadan [130]. Likewise, Bener *et al*. reported no significant increase in stroke during Ramadan [131]. Several factors, such as weather differences, number of fasting hours, degree of glycemic control, age, and duration of diabetes, may contribute to this discrepancy. All these factors, in addition to other risk factors of stroke, should be included in risk stratification.

Regarding PwDM and a recent cerebrovascular event, including transient ischemic attacks (TIAs), Comoglou and his colleagues reported that fasting Ramadan has an adverse effect on patients with ischemic stroke [128]. In another study, DM, among other factors, such as HTN and smoking, was found to be a risk factor for recurrent ischemic stroke within two years [132]. In patients with TIA, the incidence of subsequent stroke was as high as 21.5% within seven days [133], 6% after one year [134], and up to 9% within five years [135]. Moreover, Lioutas *et al.* found that despite advances in secondary prevention, the risk of stroke following TIA remained greater than the risk among TIA-free participants, and 49% of strokes occurred after 12 months from index TIA [133]. From the available data, we consider that a recent cerebrovascular event, whether stroke or TIA, is a very high risk for Ramadan fasting, at least for six months after the event, and the risk may be extended for one year according to the patient condition, whether his/her risk factors are controlled or not.

##### 2.8.2.1 Summary of Recommendations

- Recent cerebrovascular thrombotic events six months before Ramadan (stroke, TIA) are considered a very high-risk group and must not fast during Ramadan.
- PwDM and cerebrovascular events developed more than six months are considered a high-risk group and should not fast during Ramadan. If they insist on fasting, strict control of all cardiovascular risk factors is mandatory.

#### 2.8.3 Ramadan Fasting in PwDM and Peripheral Vascular Disease or Diabetic Foot

DM is a major risk factor for peripheral artery disease (PAD), with a two- to three-fold increased prevalence in PwDM compared to healthy persons [136-139]. PAD is associated with increased morbidity and reduced quality of life, especially in DM patients [140]. There are no available studies on the risk of Ramadan fasting on diabetic foot patients. However, Ramadan fasting increases the risk of diabetes complications (such as hypoglycemia and hyperglycemia) and other associated comorbidities, including CVD and PAD [97, 138, 140]. Consequently, the risk of diabetic foot is anticipated to be increased during Ramadan fasting for PwDM, particularly if the condition is aggravated by infection.

As known, Ramadan fasting for PwDM increases the risk of thrombosis and affects glycemic control [141, 142]. Both factors may augment the severity of pre-existing PAD. Therefore, chronic limb-threatening ischemia (CLTI), critical limb ischemia (CLI), acute limb ischemia, or diabetic foot infection are considered very risky during Ramadan. On the other hand, less severe conditions, including compensated CLI (due to atherosclerosis, Buerger’s disease, or arthritis) and non-infected diabetic foot ulcers, are considered less risky.

Critical limb ischemia, non-healing ulcers, and limb amputation resulting in physical disability are major complications of PAD [143]. Progressive limb gangrene, rapidly enlarging wounds, or persistent ischemic rest pain are red flags for limb ischemia and indicate the necessity for limb revascularization (through bypass or endovascular surgery) [140]. This very high-risk group of PwDM should undergo urgent intervention, including intensive conservative therapy, revascularization, or amputation.

Furthermore, diabetes is associated with a high risk of deep venous thrombosis (DVT) [144]. In addition, fasting increases the risk of thrombosis due to dehydration [145, 146]. Unfortunately, there is not sufficient data on the safety of fasting in Ramadan for PwDM and recent DVT (less than three months).

##### 2.8.3.1 Summary of Recommendations

- Patients with acute limb ischemia or diabetic foot infections are categorized as a very high-risk group and must not fast during Ramadan.
- Patients with CLI and CLTI are considered among the very high-risk group and must not fast during Ramadan.
- Patients with compensated CLI, such as atherosclerotic, Buerger's disease, arthritis, and non-infected diabetic foot ulcer are considered among the moderate-risk group and can fast with medical assistance during Ramadan.
- Patients with recent DVT (within three months) are considered a very high-risk group and must not fast during Ramadan.

### 2.9 Risk Stratification in Patients with Microvascular Complications

#### 2.9.1 Risk Stratification for PwDM and Associated Chronic Kidney Disease (CKD)

There are a lot of controversies regarding Ramadan fasting in diabetes associated with CKD and kidney transplant recipients, with a paucity of strict guidelines that help nephrologists in both issues.

According to the recently published guidelines, CKD stages 4 and 5 patients (including dialysis treatment) are at very high risk, while CKD stage 3 patients are at high-risk and all are exempted from fasting [1, 3]. However, these guidelines have not clarified the recommendations regarding certain clinical situations, including the importance of proteinuria in the classification of CKD and the stability of kidney function before Ramadan fasting.

##### 2.9.1.1 Fasting During Ramadan of CKD Patients

Generally, stages 4-5 CKD and hemodialysis (HD) patients are advised not to fast during Ramadan because they are susceptible to dehydration during long fasting hours and are at risk for fluid overload due to increased fluid intake when breaking fast [147]. Patients with CKD exposed to prolonged fluid deprivation during fasting are at potential risk of superimposed acute kidney injury (AKI).

Another point to be considered in fasting patients with diabetes and CKD is the potential for derangements in their drug therapy schedules and complications pertaining to such changes, as well as the increased susceptibility to blood glucose variability and increased risk of hypoglycemia unawareness. Adverse cardiovascular events during fasting are expected to occur more frequently in the presence of comorbidities, particularly CVD [148].

According to the 2022 KDIGO classification of CKD, stages 3-5 are considered at a high to very high risk of worsening prognosis, while CKD stages 1 and 2 are considered at a high risk of worsening prognosis if they have severely increased albuminuria (ACR > 300 mg/g creatinine) [149]. Patients with nephrotic syndrome and hypoalbuminemia are at increased risk of developing DVT, particularly if the serum albumin is less than 2.5 g/dl [147]. Moreover, moderately elevated albuminuria is strongly related to an increased risk of incident ischemic stroke, myocardial infarction, and all-cause mortality among individuals with T2D who lack overt CVD [150]. Accordingly, moderate to severe albuminuria should be considered an important determinant factor in risk stratification for Ramadan fasting.

In PwDM and CKD with pre-existing CVD, there may be a high risk of major adverse cardiovascular events during fasting, which is predicted by an early rise of serum creatinine. Baseline eGFR should be stable over the months prior to issuing any guidance statements on fasting to exclude the presence of progressive acute, acute on top of chronic events, and/or a resolving AKI [148].

On the other hand, a rising serum creatinine level during fasting is a marker of AKI and has been shown to correlate to cardiovascular and kidney events, particularly during fasting. Therefore, serum creatinine and eGFR should be monitored at least once weekly to detect any decline in eGFR and to guide patients with CKD about being safe to continue fasting [151].

Lastly, fasting is a risk factor for hyperkalemia, particularly in PwDM and CKD with type 4 renal tubular acidosis (RTA) or receiving RAAS blockers. Furthermore, RAAS blockers and diuretics are potential risk factors for AKI and electrolyte derangements in fasting patients with CKD [152].

##### 2.9.1.2 Fasting During Ramadan in PwDM and Kidney Transplantation

Fasting in kidney transplantation patients carries a risk of worsening graft function, either secondary to fluid deprivation in the fasting hours or due to the inability to regularly take their immunosuppressive medications at their proper time [153].

When looking at the literature to evaluate the studies on fasting during Ramadan in kidney transplantation recipients, we have found that despite their final conclusion that fasting during Ramadan is safe and does not affect graft function, there are a lot of drawbacks to these studies. The majority of them have been conducted during Ramadan, which occurs during cold seasons, with fasting hours ranging from 10 to 12 hours; however, there is limited evidence regarding Ramadan fasting during warm seasons. For this reason, the findings may not be generalizable to hot seasons; therefore, the safety of fasting during those periods is not guaranteed. Most of these studies were observational, and they did not address the patients' adherence to the 30-days fasting protocol, which may impede the discussion of the results.

One possible issue with fasting in unwell individuals is medication adherence. In a study on 750 people fasting throughout Ramadan who were on prescription medicines for their diseases, 10% did not take their medications as advised [154]. Another research on 81 fasting patients found that 37 changed their prescription dosage patterns, 35 skipped doses, and 4 took all of their prescriptions as usual after breaking the fast [122].

Defining specific allograft function values as safe levels for Ramadan fasting is also extremely important because none of the published evidence specifies renal function levels for safe fasting. Furthermore, most kidney transplantation patients do not have an eGFR greater than 60 mL/min.

In studying the effect of Ramadan fasting on eGFR in kidney transplantation recipients, Qurashi *et al.* [155] found a decline in eGFR from 75.6 ± 29.2 to 72.2 ± 29.7 mL/min. Besides, Hejaili *et al.* [156] reported a decline in eGFR from 75.6 ± 29.2 to 70.2 ± 28 mL/min following Ramadan fasting. Moreover, some studies have reported adverse effects due to cyclosporine toxicity, acute rejection episodes, and urinary infections [157]. Although the number of patients experiencing deteriorating eGFR and other complications during Ramadan fasting is not statistically significant, the risk is still present.

None of the published studies have examined long-term follow-up of graft function in those patients following Ramadan fasting. In addition, none of the published studies have targeted the diabetic kidney transplantation recipient group.

#### 2.9.2 Summary of Recommendations

- All patients with CKD stages 3b, 4, and 5 (eGFR ≤ 45 ml/min calculated by CKD-EPI creatinine 2021 equation) are very high-risk groups and must not fast.
- CKD stage 3a (eGFR 45-59 ml/min) in the presence of ACR ≥ 300 mg/g creatinine is considered a very high-risk group and must not fast.
- CKD stages 1-2 (in the presence of ACR ≥ 300 mg/g creatinine but less than 3000 mg/g) are considered high-risk groups and should not fast.
- CKD stage 3a with/without ACR < 300 mg/g creatinine is considered a moderate-risk group and can fast with caution, provided serum creatinine is stable in the last three months prior to Ramadan.
- Patients with AKI are categorized among the very high-risk group and must not fast.
- Patients with nephrotic range proteinuria (ACR ≥ 3000 mg/g creatinine) are considered a very high-risk group and must not fast.
- All kidney recipients are considered a high-risk group and should not fast.
- Kidney recipients with stable graft function whose immunosuppressive drug doses and frequency will not be disrupted by fasting, *e.g*., in winter rather than summer, are considered a moderate-risk group and are allowed to fast with caution, provided that they are insisting on fasting.
- Patients with known electrolyte abnormalities (type 4 RTA, patients on ACEIs/ARBs, diuretics, and mineralocorticoid receptor antagonists), and also patients at risk of dehydration, *e.g*., those on diuretic therapy, are considered a high-risk group and should not fast.
- For safe fasting, patients who insist on fasting are advised to:
  - Have rest during fasting time.
  - Avoid dehydration during fasting.
  - Take care of alarming symptoms, such as gaining > 2 kg, pedal edema, shortness of breath, and/or lethargy.
  - When breaking fast, a high-potassium and phosphorus diet should be avoided with an adequate amount of fluid.
  - Check serum creatinine at least once weekly during fasting and advise against fasting if serum creatinine levels increase by 30% above the baseline values.

#### 2.9.3 Risk Stratification for Diabetic Retinopathy (DR)

Data on the impact of Ramadan fasting on the diabetic eye, especially DR, are limited and with conflicting results. In a study that included 64 PwDM who fasted during Ramadan, researchers found significant improvement in electroretinogram (ERG) after Ramadan [158]. This may be due to fluid intake limitation, which decreases retinal exudate despite increasing the risk of thrombosis [158]. Blood glucose variability was reported to increase the risk and rate of progression of DR in both T1D and T2D [159], and glucose variability was initially increased in PwDM fasting during Ramadan [160]. Neurodegeneration and structural damage to the retina can develop over time due to DR-related glucose fluctuations, leading to eventual blindness in people with T1D [161]. Moreover, proliferative diabetic retinopathy (PDR) is associated with an elevated risk of CVD regardless of other known cardiovascular risk factors [162]. Taking into consideration the above-mentioned data, we agree with the ADA/EASD recommendation of categorizing PwDM and sight-threatening retinopathy, which includes severe non-proliferative diabetic retinopathy (NPDR), PDR, and vascular occlusive retinopathy as a high risk for Ramadan fasting [1].

#### 2.9.4 Risk Stratification for Diabetic Neuropathy (DN)

As for diabetic peripheral neuropathy (DPN), one study showed a deterioration in PwDM fasting during Ramadan [158]. In addition, DPN is considered a risk factor for developing CVD in PwDM [1]. The fluctuation of glucose levels is a significant risk factor contributing to the morbidity of DPN and diabetic autonomic neuropathy (DAN) [163]. Glucose fluctuations have been revealed to possibly be a contributing factor in the development of DPN, as well as cardiovascular autonomic neuropathy, in a number of retrospective longitudinal studies conducted on PwDM [164,165]. Also, diabetes-related neuropathy can compromise cardiac autonomic regulation [161].

DAN is a serious and frequent complication of DM. Although it has been associated with an increased risk of cardiovascular mortality, the significance of DAN has not been adequately acknowledged. Heart rate variability, a measure of cardiovascular autonomic function, has a substantial correlation (*i.e*., relative risk is doubled) with silent myocardial ischemia and mortality [166].

##### 2.9.4.1 Summary of Recommendations

- Patients with sight-threatening retinopathy are in high-risk groups and should not fast during Ramadan.
- Patients with advanced peripheral neuropathy and autonomic neuropathy are in high-risk groups and should not fast during Ramadan.

### 2.10 Risk stratification for PwDM and Associated Comorbidities (COVID-19)

The impact of acute illnesses, such as COVID-19, on the glycemic control of PwDM can be significant. Several studies have reported an increased risk of critical illness and mortality in PwDM who have contracted COVID-19 compared to non-diabetic individuals [167, 168]. Stress from the viral infection can lead to changes in insulin sensitivity and other factors that can cause hypoglycemia or hyperglycemia [169]. PwDM should work with their healthcare provider to closely monitor their glucose levels during viral infection and adjust regimens as needed. Moreover, the lockdown associated with the COVID-19 pandemic has significantly affected hospital visits, medication availability, healthcare access, and routine care of PwDM. During the COVID-19 pandemic, some studies observed no deterioration of glycemic control in PwDM [170, 171], while others indicated a worsening of glycemic control and weight increase [172, 173].

Acute illnesses, like COVID-19, require extensive management protocol, including good hydration, nutritional supplements, analgesics, and sometimes corticosteroids. This protocol could be affected by Ramadan fasting, leading to the deterioration of general health. Furthermore, some studies have demonstrated a significant deterioration in the glycemic control of PwDM fasting during Ramadan while they have contracted COVID-19 [174,175]. Therefore, the panel agreed that PwDM must not fast during acute illness (fever, COVID-19, diarrhea, *etc*.). Moreover, the restriction of fasting should extend to patients who have elevated D-dimer levels or are treated by anticoagulants due to the possibility of thrombotic complications after COVID-19 infection.

### 2.11 Summary of Recommendations

- Patients with acute COVID-19 infection are categorized as very high-risk and must not fast during Ramadan.
- During the post-COVID-19 phase, PwDM, unless controlled, are among the high risk and should not fast during Ramadan.
- In the post-acute phase, restriction of fasting should extend in certain individuals, such as those with severely uncontrolled diabetes, patients receiving corticosteroids, and patients treated with anticoagulants in acute illness. Also, patients who have disabling post-COVID-19 sequelae are considered high-risk and should not fast during Ramadan.
- Due to the rapidly evolving pattern of COVID-19 infection and the development of new variants with various sequelae, the recommendations are liable to modifications in future studies.

### 2.12 Risk Stratification for PwDM and Diabetes-related Psychiatric Complications

Diabetes is complicated by multiple psychological states, such as depression, anxiety, eating disorders, diabetes distress, and cognitive impairment that have an impact on self-care and should be screened in PwDM [176].

Ramadan fasting for PwDM and mental illnesses is closely related to the severity and chronicity of their conditions, in addition to the properties of medications received. Moreover, during Ramadan, abnormal sleep patterns can exacerbate the severity of mental illness [177, 178]. This highlights the importance of collaboration between mental health professionals and diabetologists.

ADA position statement 2016 recommended referral of PwDM to a mental health specialist for evaluation and treatment if they have the following conditions, such as depression, anxiety, eating disorders, and patients with persistent diabetes distress despite tailored diabetes education [176].

#### 2.12.1 Summary of Recommendations

- The panel agreed to refer PwDM and psychiatric disorders to mental health providers to evaluate the safety of Ramadan fasting.
- Collaborative care between both mental health providers and diabetologists is recommended to optimize both mental health and diabetes management and to ensure patients’ safety during Ramadan.

### 2.13 Risk Stratification: General Considerations

Pre-Ramadan counselling of PwDM who want to fast during Ramadan is very important for safe fasting. Giving advice concerning physical activity, nutrition, medication modification, when to break fast, and stressing the importance of SMBG is mandatory [2].

Hyperglycemia is associated with the progression of chronic complications [179] and increased risk of dehydration and thrombosis [144]. The degree of glycemic control has been considered an important determinant in risk stratification of PwDM who intend to fast during Ramadan. In the ADA/EASD statement 2020, PwDM were categorized into very high-risk or high-risk categories for fasting if average fasting or premeal glucose > 300 mg/dl and 150-300 mg/dL, respectively. Also, HBA1c levels> 10% or from 8-10% were considered very high-risk or high-risk categories, respectively. Moderate and mild-risk individuals are those with A1c < 8% and < 7%, respectively [1]. From the practical point of view, the expert panel adopted the ADA/EASD risk stratification as regards glycemic control.

In contrast to healthy individuals, Ramadan fasting in PwDM is associated with wide intra- and inter-individual variability in glucose level as evidenced by CGM, with a rapid increase in blood glucose after Iftar, most probably due to the carbohydrate-rich foods typically taken at this meal [180]. Coupled with education, real-time glucose monitoring can improve glycemic control and decrease hypoglycemia frequency during Ramadan in T1D subjects as compared to post-Ramadan [181, 182].

In pre-Ramadan, structured education is crucial and should discuss the importance of blood glucose monitoring in safe fasting with PwDM who intend to fast, especially insulin-treated people and those on SUs [183]. The selection of the method of blood glucose monitoring depends on the availability and cost, among other factors. This may be conducted with SMBG or real-time glucose monitoring (CGM or flash blood glucose testing). To the best of our knowledge, until 2021, the relative impact of blood glucose monitoring has not been addressed in risk stratification in PwDM who intend to fast. In DAR 2021, the monitoring of blood glucose has been considered in the scoring system of risk stratification for fasting Ramadan in PwDM, but it needs to be more simplified [3].

Another point to be considered is the length of fasting hours, which varies in different regions of the world and has not been considered in risk stratification until added in the DAR 2021 risk stratification for Ramadan fasting [3]. The panel agreed that long fasting hours may be associated with a higher risk of hypoglycemia during the day, especially in insulin or SUs-treated individuals.

The temperature, both the climate and work environment, has not been considered previously in risk stratification of PwDM who intend to fast. The high temperature and the longer fasting hours may be associated with an increased risk of dehydration, feeling unwell, and postural hypotension, especially in people with autonomic neuropathy. Moreover, symptomatic hyperglycemia with polyuria in volume-depleted subjects increases blood viscosity and thrombogenicity [184]. The panel advocates that each region should take into consideration the heat index (both temperature and humidity) in risk stratification. Further studies are needed to define the exact estimate of heat index for risk stratification. Regarding hard-labour workers, they were considered in the high-risk category for Ramadan fasting in DAR 2017 [2] and the very high-risk category in ADA/EASD consensus 2020 [1]. Again, this group has been underestimated in DAR 2021 [3]. The panel considers hard-labour workers a high-risk category for Ramadan fasting in regions or occupations with high temperatures and long fasting hours.

#### 2.13.1 Summary of Recommendations

- PwDM and HBA1c> 10% and hyperglycemic symptoms and/or premeal blood glucose > 300 mg/dl are considered a very high-risk group and must not fast during Ramadan.
- PwDM and HBA1c< 7% are considered a low-risk group and can fast safely during Ramadan.
- PwDM who do hard physical work receiving more than one insulin injection daily are considered a very high-risk group and must not fast during Ramadan.
- PwDM who do hard manual work are considered an intermediate risk group and can fast during Ramadan under medical supervision.
- PwDM and prolonged exposure to hot climate are considered a moderate risk group and can fast during Ramadan under medical supervision.
- PwDM who have a fasting duration >16 hours are considered an intermediate-risk group and can fast during Ramadan under medical supervision.
- PwDM and noncompliance to SMBG are considered an intermediate risk group and can fast during Ramadan under medical supervision.

## CONCLUSION

In conclusion, comorbidities can negatively impact PwDM, while impaired glycemic control can fasten the progress of comorbidities and increase the risk of associated mortality. Thus, in patients who intend to fast during Ramadan, risk stratification is crucial to optimize patient outcomes and prevent serious complications. The present AASD consensus provides a comprehensive risk assessment of PwDM according to the nature and severity of comorbidities. Patients with stable conditions and controlled DM may fast under medical assistance, while a wide range of conditions have been categorized as very high-risk groups, necessitating exemption from obligatory fasting. Future research is warranted due to the controversial literature regarding the impact of fasting on associated comorbidities.

## AUTHORS’ CONTRIBUTION

All authors were involved in data collection and literature search. All authors were involved in risk stratification, tabulation, and voting.

## Figures and Tables

**Table 1:** Risk stratification of PwDM who intend to fast during Ramadan.

**PwDM Group**	**Very High Risk**	**High Risk**	**Moderate Risk**	**Low Risk**
**Type of treatment** **A. Oral therapy**	-	-	-	Individuals on treatment with diet and other lifestyle measures
	-	-	Individuals on second-generation SUs* (except for gliclazide MR)	Individuals with diabetes receiving metformin, alpha-glucosidase inhibitors, thiazolidinediones, and DPP-4 inhibitors
	-	-	Individuals on prandial glucose regulators (glinides)	Individuals on stable doses of GLP1-RAs at least 4-6 weeks before Ramadan
	-	-	-	Individuals on gliclazide MR
	-	-	-	Individuals on SGLT-2 inhibitors
**B. Injectable therapy**	T2D patients on multiple daily insulin injections	T2D patients on two doses of premixed insulin	Treatment with basal insulin or a single daily dose of premixed insulin (both human and analogue)	-
	-	-	Individuals on FRC (basal insulin + GLP1 RA)	-
**People with special situations** **A. Pregnancy and lactation**	Pregnant ladies with diabetes, whether T1D, T2D, or gestational diabetes	-	-	-
	Lactating women with diabetes on insulin therapy	-	Lactating women with diabetes who are not taking insulin	-
**B. Old age**	Frail older people	Older people with poor health or having associated comorbid conditions	Older people >75 years who do not have any other comorbidities	-
	Elderly with cognitive disorders and dementia	-	-	-
**C. T1D**	Poorly controlled T1D individuals or those with comorbidities and/or chronic complications	Well-controlled T1D individuals	Well-controlled T1D individuals using insulin pump and/or CGM	-
**Acute complications**	Hypoglycemia unawareness	-	-	-
	Recurrent or unexplained hypoglycemia in the last three months		-	-
	DKA in the last three months before Ramadan	-	-	-
	Hyperosmolar coma in the last three months before Ramadan	-	-	-
**Macrovascular complications** **A. Cardiovascular disease**	Recent vascular thrombotic events six months before Ramadan (acute coronary syndrome) or revascularization procedures (PCI or CABG)	CCS and stable ASCVD	Mild/moderate valvular heart disease	-
	Severe valvular heart disease or prosthetic heart valves	-	Uncontrolled hypertension	-
	Severe CHF, acute HF, or ejection fraction <35%	-	-	-
	Hypertensive urgency or emergency	-	-	-
	Poorly controlled arrhythmia (including the long-QT syndrome)	-	-	-
**B. Cerebrovascular disease**	Recent cerebrovascular thrombotic events six months before Ramadan (stroke or TIA)	Cerebrovascular events developed > 6 months	-	-
**C. Peripheral vascular disease or diabetic foot**	Acute limb ischemia	-	Compensated CLI, such as atherosclerotic, Buerger's disease, and arthritis	-
	CLI and CLTI	-	Non-infected diabetic foot ulcer	-
	Diabetic foot infections	-	-	-
	Recent DVT (within three months)	-	-	-
**Microvascular ** **complications** **A. CKD**	Acute kidney injury	-	-	-
	Hemodialysis	-	-	-
	CKD stages 3b, 4, or 5	CKD stages 1-2 with ACR 300-3000 mg/g creatinine	CKD stage 3a with/without ACR < 300 mg/g creatinine	-
	CKD stage 3a + ACR >300 mg/g creatinine	Kidney transplant recipients	Kidney transplant recipients with stable graft function (in certain circumstances^#^)	-
	Nephrotic range albuminuria (ACR ≥ 3000 mg/g creatinine)	Electrolyte abnormalities (type 4 RTA, patients on ACEIs/ARBs, diuretics, and mineralocorticoid receptor antagonists)	-	-
	-	Individuals at risk of dehydration (patients on diuretic therapy)	-	-
**B. Diabetic ** **retinopathy**	Sight-threatening diabetic retinopathy	Severe NPDR, PDR, and occlusive vascular retinopathy	-	-
**C. Diabetic** **Neuropathy**	-	Advanced DPN or advanced DAN	-	-
**Associated comorbidities (COVID-19)**	During acute illness (fever, COVID-19, diarrhea, *etc*.)	Post-COVID-19 complications	-	-
**General considerations**	HbA1c >10% with hyperglycemic symptoms and/or premeal blood glucose > 300 mg/dl	HBA1c 8 - 10% with premeal blood glucose 150–300 mg/dl	HbA1c <8%	HbA1c<7%
	Hard physical work receiving more than one insulin injection daily	-	Hard manual work	-
	-	-	Prolonged exposure to hot climate	-
	-	-	Fasting duration >16 hours	-
	-	-	Noncompliance to SMBG	-

**Abbreviations**; SUs: Sulphonylureas, MR: Modified release, DPP-4 inhibitors: Dipeptidyl peptidase-4 inhibitors, GLP1-RAs: Glucagon-like peptide-1 receptor agonists, SGLT-2 inhibitors: Sodium-glucose co-transporter-2 inhibitors, T2D: Type 2 diabetes, FRC: Fixed ratio combination, T1D: Type 1 diabetes, CGM: Continuous glucose monitoring, DKA: Diabetic ketoacidosis, PCI: Percutaneous coronary intervention, CABG: Coronary artery bypass graft, CCS: Chronic coronary syndrome, ASCVD: Atherosclerotic cardiovascular disease, CHF: Congestive heart failure, HF: Heart failure, TIA: Transient ischemic attack, CLI: Critical limb ischemia, CLTI: Chronic limb-threatening ischemia, DVT: Deep vein thrombosis, ACR: Albumin-creatinine ratio, CKD: Chronic kidney disease, RTA: Renal tubular acidosis, ACEIs: Angiotensin-converting enzyme inhibitors, ARBs: Angiotensin receptor blockers, NPDR: Non-proliferative diabetic retinopathy; DPN: Diabetic peripheral neuropathy, DAN: Diabetic autonomic neuropathy, PDR: Proliferative diabetic retinopathy, SMBG: Self-monitoring blood glucose.

**Note:** *Glibenclamide (among second-generation SUs) must be changed before Ramadan. # See text.

**Very high risk = must not fast; High risk = should not fast; Moderate risk = fast under medical supervision; Low risk = can fast safely.**

In the presence of two high-risk factors, the candidate will be considered very high risk.

In the presence of five moderate risk factors, the candidate will be considered among the very high-risk.

In the presence of one high-risk factor, together with three risks from the moderate group, the candidate will be considered very high-risk.

In the presence of four moderate risk factors, the candidate will be considered among the high-risk.

## LIST OF ABBREVIATIONS

PwDM: People with Diabetes
AASD: Association for the Study of Diabetes and Metabolism
HCPs: Healthcare Providers
DM: Diabetes Mellitus
SMBG: Self-monitoring Blood Glucose
CVD: Cardiovascular Disease
